# Missing Something? Comparisons of Effectiveness and Outcomes of Bariatric Surgery Procedures and Their Preferred Reporting: Refining the Evidence Base

**DOI:** 10.1007/s11695-020-04659-1

**Published:** 2020-05-15

**Authors:** Walid El Ansari, Kareem El-Ansari

**Affiliations:** 1 Department of Surgery, Hamad General Hospital, Hamad Medical Corporation, 3050 Doha, Qatar; 2 College of Medicine, Qatar University, Doha, Qatar; 3 Schools of Health and Education, University of Skovde, Skovde, Sweden; 4 Hamad General Hospital, Hamad Medical Corporation, 3050 Doha, Qatar

**Keywords:** Post-operative, Bariatric surgery, Physical activity, Diet, Effectiveness, Outcomes, Preferred reporting

## Abstract

Comparisons of effectiveness of bariatric surgery (BS) procedures encompass weight loss, metabolic/clinical outcomes, and improvements or worsening of comorbidities. Post-operative physical activity (PA) and diet influence such outcomes but are frequently not included in comparisons of effectiveness. We assessed the value and necessity of including post-operative PA/diet data when comparing effectiveness of BS. Including post-operative PA/diet data has significant benefits for BS and patients. The paper proposes an explicit preferred reporting system (Preferred REporting of post-operative PHYsical activity and Diet data in comparisons of BS effectiveness: PRE-PHYD Bariatric). Including post-operative PA/diet data could result in more accurate appraisals of effectiveness of BS procedures. This could translate into better ‘individualized’ BS by achieving a better ‘fit’ between patient and procedure.

## Introduction

Comparisons of short-, medium-, or long-term effectiveness and outcomes of various bariatric surgery (BS) procedures, and comparisons of effectiveness and outcomes of BS vs no surgery usually encompass a range of variables. Important outcomes include safety (e.g., adverse events, operative mortality, perioperative complications, readmissions, long-term reoperation rates) or other relevant indicators (e.g., length of hospital stay) [1–7]. In addition to such variables, the indicators of effectiveness employed for comparisons frequently comprise three main components. These components include various anthropometric weight loss (WL) measures [e.g., WL, BMI, %BMI change, % excess BMI loss, percent total weight loss (%TWL), percent excess weight loss (%EWL), others)], a range of biochemical/ metabolic/clinical outcomes (FBG, HA1c, lipids, others), and improvements or worsening of comorbidities (e.g., type 2 diabetes mellitus) that are frequently gauged based on the post-BS levels of biochemical/clinical parameters [4, 7, 8]. However, the extent and quality of a patient’s post-operative (post-op) physical activity (PA) and/or diet can both influence these anthropometric WL and biochemical/ clinical outcomes, directly (e.g., via WL) or indirectly (e.g., via improvements in insulin sensitivity, metabolic health). It therefore seems important to include information on post-op PA and diet in analyses of comparisons of effectiveness of various BS procedures for a more ‘realistic’ appraisal. In practice, this seems not to be always the case. These considerations inspired the current review.

## Materials and Methods

### Research Questions

The current review explored four related questions: (1) Do post-op PA/diet need to be accounted for in comparisons of effectiveness of different BS procedures?; (2) If yes, are post-op PA/diet currently included in comparisons of effectiveness?; (3) How could post-op PA/diet be included in comparisons of effectiveness?; and, (4) What are the preferred reporting methods for explicitly elucidating whether post-op PA/diet data were collected and/or included in comparisons of effectiveness, and their emergent findings?

### Information Sources

We searched electronic databases including PubMed, MEDLINE, Embase, CINAHL, Web of Science, Scopus, and Google scholar for published articles of comparisons of outcomes of various BS procedures or comparisons of outcomes of BS procedures with medical management relevant to answering these research questions.

### Keywords and Search Terms

We used the keywords “bariatric surgery” [in Title/Abstract]. The medical subject headings (MeSH) terms used were bariatric surgery [All Fields] AND (“effectiveness”[MeSH Terms]; bariatric surgery [All Fields] AND (“outcomes”[MeSH Terms]; bariatric surgery [All Fields] AND (“comparison”[MeSH Terms]; bariatric surgery [All Fields] AND (“activity AND nutrition”[MeSH Terms]; bariatric surgery [All Fields] AND (“postoperative” AND exercise“[MeSH Terms]; bariatric surgery [All Fields] AND (“postoperative” AND diet” [MeSH Terms].

### Inclusion Criteria and Study Selection

Study design: (1) Original studies.

Language: (2) Published articles in English language.

Time Period: (3) Original studies published from 01 January 1990 through 28th February 2020.

Interventions: (4) Published articles that assessed bariatric surgery.

Participants: (5) Published articles enrolling patients of any age, gender, and ethnicity anywhere in the world.

### Exclusion Criteria

1. Studies that did not include outcomes or comparisons.

### Data Items Extracted

The review assessed whether post-op PA/diet was associated with WL after BS; examples of non-inclusion of post-op PA/diet in comparisons of effectiveness; reasons why post-op PA/diet need to be included in comparisons of effectiveness; whether post-op PA/diet is used as ‘process’ or as ‘outcome’ variables in comparisons of effectiveness; and, examples of tools that collect data on post-op PA/diet among bariatric patients and their related challenges.

Based on the emergent findings, the review proposed a way forward for a preferred reporting of post-op PA/diet in comparisons of effectiveness of BS procedures.

## Results

### Associations Between Post-Op PA and WL After Bariatric Surgery

Evidence suggests that post-op PA is associated with WL after BS. For patients who underwent primary Roux-en-Y gastric bypass (RYGB) or sleeve gastrectomy (SG), change in leisure activity at 24 months was positively associated with %TWL at 24 months, where patients with higher improvements in leisure activity had better WL [9]. Likewise, structured exercise resulted in additional improvements in insulin sensitivity after RYGB, but higher amounts of exercise were needed to achieve additional WL [10]. Patients achieving successful WL post-surgery were more likely to report higher PA than those with no successful WL [11]; higher post-op PA was positively associated with greater WL over the short term [12]; and, post-op, PA was associated with a higher likelihood of lower BMI [13]. Furthermore, post-BS exercise may provide additional improvements in metabolic health compared with surgery-induced WL alone [14]; adherence to post-BS exercises is a good prognostic factor for significant WL [15], and PA was associated with %EWL [11]. Likewise, after BS, weight regain (WR) is associated with low PA and is easy with high-calorie food, so it is necessary to modify lifestyle to combine anaerobic and aerobic exercises [16–18].

Despite all the above, there is a dearth of data on the effects of exercise on WL and WL maintenance after BS [14]. Although inclusion of PA and exercise in clinical follow-up schedules greatly benefits BS patients since this leads to greater improvements in body composition, bone mineral density, muscle strength, and fitness [19], participants’ activity pre- and post-surgery showed that their PA levels pre- and post-surgery did not differ [20]. These findings suggest the need to measure PA post-BS and incorporate it in the analysis in order to realistically compare the effectiveness of various BS procedures.

### Associations Between Post-Op Diet and WL After Bariatric Surgery

Binging and grazing eating patterns after BS are associated with poor outcomes [15]. Post-BS, morbidly obese people achieve more WL if they follow, e.g., a Mediterranean diet [21]. A WL diet is very important after WL surgery [22], and as weight is easily gained with high-calorie food after BS, it is necessary to control the diet [16]. Adherence to post-BS nutritional plans is considered a good prognostic factor for significant WL [15], and WR after BS is associated with poor dietary adherence [17, 18]. Recently, a systematic review suggested that BS can reduce energy intake but can result in unbalanced diets, inadequate micronutrient and protein intakes, and excessive fat intake which contribute to WR [23]. Others found that six maladaptive eating behaviors accounted for a highly significant portion of post-RYGB patients’ poor self-reported dietary adherence, proposing that research is needed to assess the associations between maladaptive eating behaviors and BS outcomes [23].

The post-op quality of diet is also important, as various BS procedures might be associated with different post-op diet preferences. A year after surgery, RYGBP patients ate significantly less carbohydrates and more lipids and had higher daily cholesterol intake than the SG patients [24]. In/direct measurements of eating behavior suggest that food selection changes after BS, with reduced preference for food high in sugar and fat [25]. These findings suggest that researchers need to assess food intake and its quality and incorporate it in the analysis to realistically compare the effectiveness of various BS procedures.

### Why Include Post-Op PA and Diet in Analyses of Comparisons of Outcomes Between Various Bariatric Procedures?

The above evidence proposes that it could be appropriate to include post-op PA/diet data in the analyses of comparisons of effectiveness between various BS procedures, or in comparisons of effectiveness of BS vs no surgery. Long-term dietary control and PA can help patients achieve optimal WL [16]. Hence, a given BS procedure might be assessed to be more effective than another because (a) it is ‘truly’ more effective or, (b) post-op, patients who had undergone a given BS procedure had, either by chance or due to certain characteristics, higher PA/exercise levels, adhered to better diets/healthier eating patterns, or both, factors that could contribute to their better observed outcomes compared to patients who undertook another BS procedure. Hence, the concern is that when post-op PA and/or diet are not accounted for in the analyses, then findings and conclusions about the effectiveness of different BS procedures might get ‘contaminated’ by any associations of PA/diet with the selected outcomes that are examined.

### Examples of Non-Inclusion of Post-Op PA and Diet in Comparisons of Outcomes Between Various Bariatric Procedures

Yet, the majority of studies seem not to collect or include data on these two important variables (PA and diet) that can potentially influence the gauging of effectiveness. This is regardless whether such studies are short-, medium-, or long-term comparisons of effectiveness between “variants” of one BS procedure, e.g., regular biliopancreatic limb RYGB vs. long biliopancreatic limb RYGB [26]; between two or more BS procedures, e.g., one-anastomosis gastric bypass vs. RYGB, or laparoscopic RYGB vs. laparoscopic SG [27, 28]; between primary and revisional BS, e.g., primary vs. revisional gastric bypass [7]; or between BS procedure/s vs. no procedure (e.g., medical management) [29, 30]. Interestingly, most studies appear not to have included, in their limitations, a note that post-op PA and/or diet data were not collected and/or were not included in the analyses of comparisons of the BS outcomes [7, 26–28, 31]. This is not a preferred reporting method. However, few exceptions exist.

Some studies acknowledged the lack of control for post-op PA/diet in their analyses of comparative effectiveness of various BS procedures. A comparison of the effects of RYGB vs SG on body mass composition explicitly acknowledged its limitations which included the inability to evaluate accurate protein and macronutrient consumption or differences in PA patterns, highlighting that “while all their patients undergo similar dietary education with emphasis on daily exercise, this was not strictly controlled for” p. 454 [8].

Other studies undertook case-matched analysis. A comparison of laparoscopic very, very long limb revisional vs primary RYGB-matched patients by gender, age, preoperative/pre-revisional BMI, and diabetes [32]. Unfortunately, post-op PA/diet were not included in the analysis, despite that EWL was an examined outcome [32]. Another long-term matched comparison of adjustable gastric banding vs sleeve gastrectomy (matching criteria: age, weight, surgery date) did not include post-op PA/diet in the analysis, despite that mean total body WL was an examined outcome [6]. Likewise, research compared the efficacy of primary vs revisional laparoscopic RYGB using matched analysis (matching criteria: age, gender, preoperative BMI, follow-up period), but post-op PA/diet were not included in the analysis, despite that WL was an examined outcome [33]. In addition, given the matching, it is not clear whether the statistical analysis employed for such matched studies was the most appropriate [34], which may lead to inaccurate estimation of association between exposure and outcome [35], and hence influence the appraisal of effectiveness.

Still, others seem to have collected the requisite data. A comparison of effectiveness of RYGB vs SG vs SG with jejunal bypass collected daily diet and leisure-time exercise data by telephone interviews [16]. However, it is unclear how this post-op PA/diet data was used in the comparison of the procedures’ outcomes, despite that the appraisal of effectiveness included four anthropometric measures [16]. Perhaps the post-op PA/diet data was not useful in this study, as the authors noted that “all patients enlisted in our telephone interview followed the doctor’s advice to maintain exercise and a diet for at least 1–2 years after surgery” p. 181 [16]. Without documented variations in patients’ post-op PA/diet, the information is rendered not useful in being incorporated in the analyses. Surprisingly, the authors reported that “some patients returned to hospital for the second surgery or other ways to lose weight because of poor dietary habits or lack of exercise” p. 181 [16], suggesting that the information provided by patients was not always precise.

Very few exceptions illustrate the importance of inclusion of post-op PA and diet in the analyses. Research of WR in RYGB vs SG patients with symptoms of post-BS hypoglycemia (PBSH) was concerned about the potential confounding effect of nutritional adherence on WR [36]. Hence, the researchers explored the relationships in separate analyses stratified by level of nutritional adherence, to show that WR ≥ 10% was significantly positively associated with presence of PBSH among those less adherent to nutritional guidelines, but no association among patients with high adherence to guidelines [36]. This represents an example of better analysis and reporting.

### Post-Op PA and Diet Used as Process or Outcome Variables

When PA/diet data are available, a related point is the manner in which such data is employed. Post-op PA and diet data is frequently used as outcomes *per se* (i.e., used to compare effectiveness of various BS procedures). As an alternative, post-op PA can be used as process variables, i.e., mobilized to help verify that observed differences in BS outcomes are due to the BS itself and not actually influenced by differences in patients’ PA/diet (i.e., used to increase certainty that effectiveness of various BS procedures are actually due to the procedures and not due to an artifact).

For instance, a study collected information that could implicitly have some requisite data. A comparison of SG vs one-anastomosis gastric bypass (OAGB) measured quality of life in both groups using the Moorehead-Ardelt quality of life questionnaire II that assessed, among other things, approach to PA and interest in food [37, 38]. However, the PA and diet data were used as outcomes rather than being incorporated in the analysis to assess the potential effects of SG vs OAGB on WL ‘uncontaminated’ by post-op PA or diet [38]. Others compared primary vs revisional laparoscopic RYGB employing two questionnaires, SF 36 (focuses on aspects of life quality that include physical functioning) and Moorehead-Aldert II QLQ test (estimates areas of everyday life including PA and eating behavior) [33]. However, despite the availability of some post-op PA and diet information, and despite that effectiveness was gauged by WL and improvement of comorbidities, such post-op PA/diet data were used as outcomes rather than being incorporated in the analysis to assess whether effectiveness of the two procedures was influenced by differences in post-op PA/diet of patients in each group (i.e., not used as ‘process’ variables) [33].

### Problematizing the Equation: Inconsistencies and Synergies

Notwithstanding, relationships between post-op PA/diet on the one hand and the anthropometric WL and biochemical/clinical outcomes of BS on the other exhibit inconsistencies. After BS, individuals who increased their PA tended to lose more weight than those who maintained or decreased their PA, but the WL differences were not significant [21]. Likewise, there were no significant associations of change in sport and work activity with %TWL at 24 months for RYGB or SG [9]. PA after BS was not associated with the scale of WL [21]; PA was not a predictor of WL even if PA significantly increased after BS [39]; and, WL one year post-RYGB was not associated with self-reported or objectively measured PA [40].

Equally, post-BS, patients who achieved optimal WL consumed dessert more frequently than those with suboptimal WL [41]. Interestingly, after RYGB or SG, fat and sweet intake of adults who achieve and sustain optimal WL was similar to those who did not achieve/sustain optimal WL, but those with suboptimal WL had less PA or less healthy diets [41–44]. Synergies also exist: regular PA may be associated with other lifestyle behaviors e.g., healthy eating [45]. A lifestyle behavior does not usually occur in isolation; it is frequently associated with other behaviors, and ‘clustering’ of lifestyle characteristics is documented among normal individuals [46–48].

### Problematizing the Equation: Other Considerations

Other considerations exist. For instance, RYGB and SG decreased the hedonic evaluation of high-fat food stimuli, but this did not translate into decreased preferences for high-fat food [49]. Post-BS, patients reported changes in their flavor perception and food preferences (decreased preference for energy-dense foods, particularly, sweets, high fats) [50–52]. However, validated techniques found little/no change in patients’ ability to perceive taste or preference for energy-dense foods, suggesting that the changes in taste and food preferences could be related to changes in the rewarding value of food [52]. Further, RYGB and possibly SG might be associated with increased risk for alcohol use, supporting that some BS might alter central circuits of reward that are critical to the regulation of ingestive behavior [52].

### Data on Post-OP PA and Diet Among Bariatric Patients

Post-op adherence to exercise and diet are difficult to evaluate [53–56]. Many tools exist. In terms of PA, the Baecke questionnaire assesses the amount of time spent on leisure, work, and sport activity [9, 57]. The Bariatric Analysis and Reporting Outcome System (BAROS) and its modification [37, 58] evaluate changes in quality of life, metabolic effects, and complications after BS, but do not consider the quality of diet or PA [21]. Recently, a study used a mobile phone App to track PA among patients considered for and who had previously undergone BS [20]. Total energy expenditure can be accurately evaluated using doubly labeled water, but it is less practical for large-scale research [59, 60]. Wrist or thigh accelerometry appraise activity- and total energy expenditure and wrist-worn accelerometers objectively capture free-living PA [60–63]. Nevertheless, RYGB patients overestimated their time spent in PA to a greater extent post-surgery than pre-surgery [64]. Compared to pre-surgery, self-reported PA increased by 46.9% and 36.5% from pre- to 9 and 48 months, respectively, but accelerometer changes showed a 6.1% increase and 3.5% decrease [64].

In terms of post-op diet of BS patients, many self-report tools assess consumed food quality and tolerance, and food records of foods and beverages consumed accompanied by a picture album of food-portion sizes might enhance accuracy [24, 65, 66]. However, self-reported food frequency questionnaires suffer from underreporting, recall errors, difficulty in assessment of portion sizes, and only assess preferences or consumption frequency of foods [67–70], as opposed to a behavioral approach that incorporates measuring choices between differing food products [70].

Adherence is generally evaluated via verbal/written self-reports employing cutoffs (adherent vs non-adherent) [71] and suffer from the limitations of self-reports [72]. Metabolomics could objectively identify dietary biomarkers, where metabolite biomarkers of habitual diet are detectable in serum and urine, which is useful in large-scale investigations to categorize individuals into dietary patterns, although more evidence is required [73, 74]. After BS, adherence rates to specific dietary or PA guidelines are inconsistent across studies, with various adherence definitions and measurement methods [71]. The challenges also include drop outs, particularly with long follow up periods (medium- and long-term outcomes), and differential underreporting, where individuals with obesity or suboptimal WL were more likely to underreport fatty food/dessert consumption than those without obesity or optimal WL [75–77].

### Statistical and Analytical Considerations

**Fig. 1:**
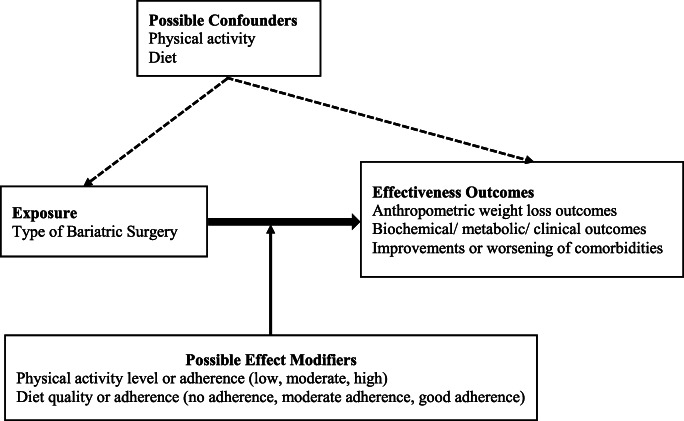
Post-operative physical activity and/or diet as potential confounders or effect modifiers for the relationship between type of bariatric surgery and effectiveness outcomes

Post-op PA or diet could act as confounders or effect modifiers. However, one will not know whether PA and/or diet is a confounder, effect modifier, or neither unless data about these variables are collected and appraisals are undertaken. Figure 1 illustrates how post-op PA/diet could influence the relationship between type/s of BS and their relative effectiveness.

A confounder is an ‘extra’ variable that the researcher did not account for; a variable, other than the independent variable of interest (e.g., type of BS), which may affect the dependent variable (e.g., WL, FBG, HA1c, others) [78, 79]. Confounding can lead to inaccurate conclusions about the association between the independent variables (type of BS) and dependent variables (WL, FBG, HA1c). At the design phase, confounding is a potential; its true presence or absence is assessed through appropriate data analyses [80]. Table 1 (section 5A) shows ways of gauging whether a variable is a potential confounder [i.e., associated with the risk factor (BS procedure) and with the outcome (WL)]. Confounding can be dealt with by controlling, matching, randomizing, or statistical control [79].

**Table 1:** Post-operative physical activity and/or diet data employed in comparisons of bariatric surgery outcomes: issues, potential solutions, and proposed preferred reporting^a^

Issue	Potential action/s	Preferred reporting^a^							
		A	B	C	D	E	F	G	H
1. No data collected on post-op PA/ diet and/or included in analyses	Highlight this point for future research direction/s	✓							
2. Case-matched analysis	Usually matching undertaken for preoperative characteristics^b^								
	Post-op PA/diet included in analysis of comparisons of effectiveness?		✓	✓	✓			✓	
	Post-op PA/diet not included in the analysis of comparisons of effectiveness?	✓							
3. Data collected specifically on post-op PA/diet	Are such post-op PA/diet data are employed as “process”^c^ vs “outcome”^d^ variables					✓			
	If data is used as process^c^ variables				✓		✓	✓	
	If post-op PA and/or diet data collected as process^c^ variables but not actually included further in the analyses of comparisons of outcomes	✓							
4. Data collected not specifically on post-op PA/diet e.g., data on quality of life parameters (SF 36, Moorehead-Aldert II, others)	Although such quality of life measures usually employed as outcomes^d^, they include information on, e.g., physical functioning, PA, and eating behavior. Such data could represent a way forward if used as process variables ^c^ to suggest some indication of post-op PA/diet data that could, if required, be included in analyses of comparisons of outcomes, using appropriate statistical technique/s. Drawbacks:		✓	✓		✓			
	(a) Same quality of life data cannot be used as process^c^ and outcomes^d^ simultaneously (b) Using some data (domain) out of a quality of life measure as process^c^ could disturb the use of whole measure as an outcome^d^. Such tools usually generate composite score across their domains despite individual scores for each domain								
	If, however, decisions are made to use such information as process variables^c^ rather than outcomes^d^ and be included in analyses of comparisons of outcomes:				✓	✓			✓
5A. Post-op PA/diet data specifically collected and included in the analyses: is any a confounder?	If post-op PA and/or diet data is included in analyses Assess a potential confounder by: (a) Formal tests of hypothesis (b) Inspect data for practically important/clinically meaningful relationship between variable and risk factor, and between variable and outcome (regardless of whether relationship is significant). If yes, variable could be a confounder (c) Appraise the measure of association before and after adjusting for potential confounder (change of ≥ 10% in estimated measure of association could suggest confounding) [81].		✓						
	If variable found to be potential confounder, confounding can be dealt with by controlling, matching, randomizing, statistical control [79]. If testing for confounding is done:		✓	✓	✓			✓	
5B. Post-op PA/diet data specifically collected and included in analyses: is any an effect modifier?	If post-op PA and/or diet data is included in analyses. For observational studies, test effect modifier by: Outcomes of BS (e.g., WL) are assessed across subgroups of patients of different PA levels and/or diet adherence (e.g., no adherence, moderate adherence, good adherence) [82] to see if differs depending on the level of a third variable (post-op PA or diet). Multivariable methods can also assess effect modification [81].		✓						
	If variable found to be effect modifier, stratify analyses by levels/ categories of effect modifier. If testing for effect modification is done:		✓	✓	✓			✓	

A: Acknowledge in limitations section the lack of inclusion of and/or control for post-op PA and/or diet in the analyses of comparisons of effectiveness. B: Explicit mention in “Materials and Methods” section and in “Statistical and Analytical Considerations” section. C: Report the specific findings of such inclusion or utilization in the “Results” section. D: Acknowledge their inclusion in the strengths of the study section regardless of whether such effects were significant or otherwise. E: Explicit mention in methods section whether post-op PA and/or diet employed as process^c^ vs outcome^d^ variables. F: Ascertain in methods section that post-op PA and/or diet data collected was actually included in analyses of comparisons of outcomes. G: Highlight in statistical analysis subsection of the methods section the test/s employed and their appropriateness. H: Report in methods section how the use of such variables as process^c^ variables might have disabled their simultaneous use as outcome^d^ variables

^a^Preferred REporting of post-operative PHYsical activity and Diet data in comparisons of BS effectiveness: PRE-PHYD Bariatric

^b^Be cautious of over-matching

^c^PA/diet data used to increase certainty that effectiveness of various BS procedures are actually due to the procedures themselves and not due to an artifact

^d^PA/diet data used to compare effectiveness of various BS procedures

An effect modifier is to identify whether the effect of a treatment (type of BS) is different in groups of patients with different characteristics (PA/ diet) [82]. Effect modification happens when the association between the exposure (BS) and outcome (WL) differs depending on the level of a third variable (post-op PA/diet) [81]. When effect modification is present, it would be misleading to compute an overall estimate of the association (between BS and WL) because the association is different for those with or without the third factor (post-op PA or diet) [81]. For observational studies, Table 1 (section 5B) depicts ways to appraise whether post-op PA or diet are effect modifiers. Multivariable methods can assess effect modification [81].

### Conclusion: Now What?

Bariatric researchers almost always include patients’ age and sex as potential confounders when comparing the effectiveness of various BS procedures. Yet, it is not clear why patients’ post-op PA and dietary practices are not considered in such analyses. There have been calls for the comprehensive measurement of outcomes in BS [6]. However, unless there is a general belief or consensus that, post-BS, all patients are considered equal in terms of their PA and/or diet practices, comparisons of short-, medium-, or long-term effectiveness of various BS procedures, and comparisons of effectiveness of BS vs no surgery are likely to remain a reflection of the effectiveness of a given procedure, probably contaminated with the consequences of the patients’ quality and extent of post-op PA/diet. Exceptions could be prospective studies with randomization of patients to BS procedures in order to generate comparable groups, which are alike in all important aspects (e.g., post-op PA/ diet) except for the intervention (type of BS) that each group receives [83–85].

Accurate assessments of post-op PA and dietary practices are not easy and will require extra efforts from patients and research teams alike, but should preferentially be attempted and included in analyses of comparisons of effectiveness of BS procedures. In refining the evidence base, Table 1 summarizes some of the issues and potential solutions for the inclusion of post-op PA and/or diet data, and proposes an explicit preferred reporting system of such undertakings in BS (Preferred REporting of post-operative PHysical activity and Diet data in comparisons of BS effectiveness: PRE-PHYD Bariatric). Without complicating the equation, should bariatric researchers find it appropriate to ‘wash out’ some of the ‘contamination’ incurred by the unique post-op PA and/or diet practices of different patients on conclusions of effectiveness, then including PA and/or diet data in the analyses might be a way forward for more valid comparisons. Should such inclusion be or not be undertaken, a preferred reporting practice (as PRE-PHYD Bariatric outlined above) would acknowledge this.

### Implications and Potential Benefits for Bariatric Surgery and Patients

Patients’ variability in post-BS WL could be to some extent, due to differences in adherence to dietary and PA recommendations, given that physiological changes acquired through surgery *per se* do not essentially bring about positive long-term effects [86, 87]. Hence, a more accurate estimate of the effectiveness of different BS procedures will provide care providers with insights of a more realistic value or range of benefits that a given BS could confer. Such information, provided to patients, could assist them in making more informed decisions about their BS.

In addition, knowledge of whether (and extent to which) effectiveness of a given BS procedure might vary across different groups of patients with different PA/diet characteristics will be key to assist surgeons in assessing patients who could more likely benefit from a given BS procedure. Patients’ comorbidities and potential risks are included in the choice of the appropriate BS [5], and potential benefits are appraised based on the patient’s medical, anatomic, and psychosocial profile [1]. Several preoperative psychological predictors were related to post-op adherence to dietary and PA recommendations, although they were not associated with WL [53].

The inclusion of post-op PA and diet in analyses of comparisons of BS outcomes and reporting such inclusion (using e.g., PRE-PHYD Bariatric) as highlighted in the preferred reporting above could provide fresh evidence about the role of these two variables in assessments of effectiveness. Should their roles be important, the emergent knowledge on the ‘net’ gains of effectiveness of BS procedures and the magnitude of influence of post-op PA and diet could advance the field. New understandings could broaden the preoperative counseling of patients to include more evidence-based perspectives of post-op PA. These could include pre-op subjective/objective appraisals of a patient’s potential motivation and ability for post-op PA, particularly that motivation is the best predictor of adherence to exercise, and that preoperative PA and planning before RYGB predicted post-op PA [53, 88–90]. Similarly, new knowledge about the interplay between a given BS and the patient’s characteristics could expand the pre-op evaluation of patients to appraise their intention of and commitment to adherence to post-op diet recommendations. Pre-op predictors of post-RYGB dietary adherence were years with dieting experience, readiness to limit food intake, and night eating tendency [53]; and pre-op predictors of WL were higher frequency of snacking pre-op, greater past WL, and younger age [53].

Adherence to dietary and PA guidelines is associated with greater WL after surgery [56], but little is known about the features that enable adherence [91]. Including post-op PA and diet data in analyses of comparisons of BS outcomes should increase our comprehension of how these two factors intervene and interact to influence the outcomes. The hope is that these new insights will assist in the counseling and assessment of patients who could more likely benefit from a given BS procedure. Simply summarized: assist surgeons towards ‘individualized’ BS by achieving a better ‘fit’ between patient and procedure. This could be a ‘game changer’ for the field.
